# Adult-onset Still’s disease presenting with aseptic meningitis: a case report

**DOI:** 10.3389/fimmu.2025.1660434

**Published:** 2025-12-16

**Authors:** Xiao-Fen Li, Amy Ker, Hong-Li Liao, Yuan Liu, James Cheng-Chung Wei

**Affiliations:** 1 Department of Rheumatology, Liuzhou People’s Hospital, Guangxi Medical University, Liuzhou, Guangxi Zhuang Autonomous Region, China; 2 School of Medicine, Chung Shan Medical University, Taichung, Taiwan; 3 Department of Medical Education, Kaohsiung Chang Gung Memorial Hospital, Kaohsiung, Taiwan; 4 Division of Allergy, Immunology and Rheumatology, Department of Internal Medicine, Chung Shan Medical University Hospital, Taichung, Taiwan; 5 Graduate Institute of Integrated Medicine, China Medical University, Taichung, Taiwan

**Keywords:** adult-onset Still’s disease, autoinflammatory, case-report, Yamaguchi criteria, tocilizumab

## Abstract

Adult-onset Still’s disease (AOSD) is a systemic autoinflammatory disorder characterized by high spiking fever, evanescent rash, arthritis, sore throat, and lymphadenopathy. The pathogenesis of AOSD remains unclear. Neurological involvement in AOSD is uncommon. This case report describes an atypical presentation of AOSD complicated by aseptic meningitis, where the true rarity lies in the unusually severe neurological involvement and associated complications, including empty sella. A 56-year-old female was admitted with a one-year history of recurrent high-grade fever, fatigue, accompanied by seizures and episode of altered consciousness lasting for over 17 hours. During the course of hospitalization, she exhibited polyarticular swelling and pain, seizures and impaired consciousness. Laboratory analysis revealed leukocytosis and markedly elevated serum ferritin levels. Brain magnetic resonance imaging (MRI) showed diffuse leptomeningeal enhancement. Infectious, demyelinating, and other etiologies were excluded based on CSF analysis and other evaluations. A diagnosis of aseptic meningitis secondary to AOSD was made. The patient was treated with cyclosporine, tocilizumab, intrathecal dexamethasone, and methotrexate, resulting in marked clinical improvement. This case highlights the importance of recognizing uncommon neurological manifestations of AOSD and underscores that severe complications such as empty sella may occur and require prompt recognition and treatment.

## Introduction

AOSD is a rare systemic autoinflammatory disorder characterized by high spiking fevers, transient maculopapular rash, arthralgia and leukocytosis (1, 2). Neurological involvement in AOSD is uncommon and has been described in only a small number of case reports. In a single-center study by Zhao et al., approximately 8% of patients with AOSD exhibited neurological manifestations, including seizures, encephalopathy, and meningitis (3). Reports focusing on central nervous system (CNS) involvement remain scarce, and large-scale clinical studies are lacking. Here, we present an atypical case of AOSD complicated by aseptic meningitis, emphasizing the unusually severe neurological manifestations and complications such as empty sella, along with a favorable response to immunosuppressive therapy.

## Case description

A 56-year-old Asian female (Han ethnicity), a farmer by occupation, with no significant past medical history, no regular medications, no family history of autoimmune or inflammatory diseases, and no history of smoking or alcohol use, was admitted due to a year-long history of recurrent fever and fatigue, later complicated by a seizure episode with altered consciousness lasting over 17 hours. Her symptoms began in early 2023 with high fever (up to 39.5°C) accompanied by sore throat and diffuse joint pain initially without swelling. The fever pattern was intermittent, and body temperature could be reduced to normal with oral antipyretics such as ibuprofen. Early joint pain involved the knees, wrists, and shoulders; initially there was no swelling, and no rash, chills, cough, dyspnea, dizziness, or headache were noted. Over time, joint swelling developed and pain gradually worsened.

In March 2023, she was hospitalized at our facility. Laboratory investigations as summarized in **Table 1** revealed anemia, elevated inflammatory markers a negative antinuclear antibody (ANA) panel, and markedly elevated ferritin levels. Bone marrow was reactive without malignancy. Ultrasound showed multiple enlarged superficial lymph nodes, and a pathological biopsy of the left axillary lymph node confirmed reactive lymphadenitis. She was diagnosed with a connective tissue disease (CTD) and discharged after fever resolution with oral prednisone 30 mg daily. At that time, AOSD was not diagnosed because she lacked hallmark features such as arthritis, the typical evanescent rash, and leukocytosis. Although an autoimmune inflammatory disorder was suspected and prednisone was initiated, she did not return for follow-up. She subsequently sought care in other departments without rheumatology consultation, resulting in a missed opportunity for early diagnosis. She was re-hospitalized in October 2023 due to recurrent fever, arthralgia, and fatigue. During this hospitalization, she was found to have persistent hyponatremia, anemia, and positive Epstein-Barr virus antibodies. Repeated bone marrow examination and PET-CT was unremarkable. She was treated for infection-related anemia with antiviral therapy, antipyretics (nimesulide), and antibiotics, and discharged after symptom relief. Her condition subsequently relapsed. In November 2023she was admitted again due to seizures, impaired consciousness, and shock. Cranial Computer Tomography (CT) suggested lacunar infarction while chest CT revealed pulmonary infection, raising suspicion of sepsis. She was admitted to the Intensive Care Unit (ICU), however, her family declined further investigations, and she was discharged against medical advice. She took prednisone 10 mg twice daily for one month, then discontinued and occasionally used traditional Chinese medicine.

**Table 1:** Laboratory investigations.

Test Item date	WBC (10^9/L)	HB (g/l)	Ferritin (ng/ml)	Serum Sodium (mmol/L)	Aldosterone 8AM (pg/ml)	Aldosterone 12PM (pg/ml)	ACTH (pmol/L)	PRL (mIU/L)	CRP (mg/L)	ESR (mm/h)
2023/03/13	8.52	86	15429.79	133.6	/	/	/	/	62.47	105
2024/03/11	17.35	77	>33511.2	121.5↓	65.23↓	88.6↓	<0.5↓	1216.8↑	70.78	73↑
2024/05/21	4.48	111	9286.75	142.2	42.82↓	68.43↓	0.85↓	916.43↑	7.36	10↑
2024/10/29	6.24	126	1315.19	143.3	156.01	282.14	3.02	516.81	0.13	7
2025/4/03	6.02	121	888.0	145.7	277.91	1003.96	4.12	414.63	0.12	15

On March 2024, she experienced facial twitching, urinary incontinence, and foaming at the mouth, without obvious limb convulsions. She regained consciousness approximately one minute later but remained unresponsive to verbal stimuli, accompanied by high-grade fever and joint swelling and pain. After stabilization in the emergency ICU, rheumatology was consulted. Given her symptoms, AOSD was strongly suspected, and she was transferred for further treatment.

Upon admission in early March 2024, the patient had a temperature of 39.2°C measured via the axilla, blood pressure of 97/64 mmHg, pulse rate of 137 bpm, respiratory rate of 24 breaths per minute, and oxygen saturation of 87% on room air. Lung auscultation revealed coarse breath sounds with moist rales bilaterally. The abdomen was soft, non-tender, and without rebounding pain. Both wrists and knees were swollen and tender, with limited range of motion. Neurological examination revealed Glasgow Coma Score (GCS) score of 12 (E3V4M5); the patient opened her eyes to verbal stimuli and appeared distressed. Pupils were equal, round (approximately 2 mm in diameter), with sluggish light reflexes. Muscle strength was symmetrically decreased (grade 4) in all limbs. Higher cortical functions were impaired, and meningeal signs were positive. Her past medical history was notable for chronic hepatitis B surface antigen carriage without prior treatment.

The laboratory results were shown in **Table 1**, indicating leukocytosis and elevated serum ferritin level and anemia. Serum electrolytes showed hypokalemia (K^+^ 3.09 mmol/L), hyponatremia (Na^+^ 128.7 mmol/L), hypochloremia (Cl^−^ 95.4 mmol/L), and hypocalcemia (Ca^2+^ 1.80 mmol/L). Myocardial enzyme panel revealed elevated lactate dehydrogenase (LDH) at 877.5 U/L. Liver function tests showed hypoalbuminemia (albumin 19.9 g/L) and mild aspartate aminotransferase (AST) elevation (44 U/L). B-type natriuretic peptide (BNP)B-type natriuretic peptide (BNP) was markedly elevated at 3921.00 pg/mL. Inflammatory markers were significantly increased, as summarized in **Table 1**., Other laboratory tests showed interleukin-6 (IL-6) at 105.00 pg/mL, and PCT at 0.645 ng/mL. Multiple blood cultures, respiratory pathogen nucleic acid tests, cytomegalovirus DNA, Epstein-Barr virus DNA and Tuberculosis screening (PPD and T-SPOT) were all negative. Immunological tests showed elevated immunoglobulin G (IgG) at 23.940 g/L. Among autoimmune liver disease antibodies, anti-SP100 antibody was positive, while rheumatoid factor (RF), anti-CCP antibody, ANCA, ANA, and the ENA-12 panel, as well as IgG4 quantification, were all negative. Endocrine testing revealed markedly decreased serum adrenocorticotropic hormone (ACTH), while other endocrine parameters were remained within normal ranges. (**Table 1**).

In March 2024, the patient’s serum osmolality was 600 mOsm/kg H_2_O. The patient had no symptoms of diabetes insipidus. Urine osmolality was within the normal range, while serum osmolality was decreased. These findings suggest that the hyponatremia may be associated with decreased aldosterone levels and adrenal (cortisol) insufficiency. HBV DNA was 9.86 ×10² IU/mL. Lumbar puncture revealed a cerebrospinal fluid (CSF) opening pressure of 140 mmH_2_O. Routine CSF analysis and bacterial culture showed no abnormalities. Metagenomic next-generation sequencing (mNGS) of CSF revealed no detectable DNA-based pathogens, resistance genes, or virulence genes, and a cell-based assay (CBA) panel of 21 autoimmune encephalitis-related antibodies was entirely negative (**Table 2**). Anti-GFAP and anti-MBP antibodies in CSF were also negative by CBA (**Supplementary 1**).

**Table 2:** CSF analysis including metagenomic pathogen screening and autoimmune encephalitis antibody panel.

A. DNA-metagenomic Analysis Result of DNA Pathogenic Microorganisms.					
Bacteria	Fungus	Virus	Parasites	Antibiotics resistance gene	Virulence gene
Not detected	Not detected	Not detected	Not detected	Not detected	Not detected

For immunocompromised or immunodeficient patients, attention should be given to the microbiota profile.

A definitive diagnosis is recommended based on a comprehensive evaluation of clinical presentation and additional laboratory findings. (A)

**B.** Autoimmune encephalitis panel testing in cerebrospinal fluid.

Items	Test methods	result	Reference value/range
20 items of autoimmune encephalitis			
Anti-glutamate receptor (NMDA type) antibody IgG	CBA method	Negative (-)	Negative (-)
Anti-glutamate receptor (AMPA1 type) antibody IgG	CBA method	Negative (-)	Negative (-)
Anti-glutamate receptor (AMPA2 type) antibody IgG	CBA method	Negative (-)	Negative (-)
Anti-leucine-rich glioma inactivation 1) antibody IgG protein 1(LG1)	CBA method	Negative (-)	Negative (-)
Anti-contact protein-associated protein 2 (CASPR2) antibody IgG	CBA method	Negative (-)	Negative (-)
Anti-y-aminobutyric acid B receptor (GABAB) antibody IgG	CBA method	Negative (-)	Negative (-)
Anti-IgLON family protein protein 5 antibody IgG	CBA method	Negative (-)	Negative (-)
Anti-peptide-like protein 6 (DPPX) antibody IgG	CBA method	Negative (-)	Negative (-)
Anti-glycine receptor 1(GlyR1) antibody IgG	CBA method	Negative (-)	Negative (-)
Anti-dopamine receptor 2 (DRD2)	CBA method	Negative (-)	Negative (-)
Anti-glutamic acid decarboxylase 2(GAD65) antibody IgG	CBA method	Negative (-)	Negative (-)
Anti-metabotropic glutamate receptor 5 (mGluR5) antibody IgG	CBA method	Negative (-)	Negative (-)
Anti-metabotropic glutamate receptor receptor 1(mGluR1) antibody IgG			
Anti-synaptophysin-3a antibody IgG	CBA method	Negative (-)	Negative (-)
Anti-y-aminobutyric acid type A receptor (GABAA) antibody	CBA method	Negative (-)	Negative (-)
Anti-Keich-like protein 11(KLHL11) antibody	CBA method	Negative (-)	Negative (-)
Anti-ganglionic AChR acetycholine receptor antibody	CBA method	Negative (-)	Negative (-)
Anti-aquaporin 4 antibody (AQP4)	CBA method	Negative (-)	Negative (-)
Anti-myelinated oligodendrocyte glycoprotein antibody (MOG)	CBA method	Negative (-)	Negative (-)
Anti-GFAP antibody	CBA method	Negative (-)	Negative (-)

**Figure 1:**
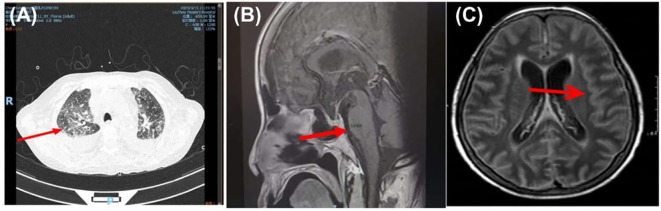
Radiological findings of the patient. **(A)** Chest CT showing bilateral pulmonary infiltrates (red arrow). **(B)** Sagittal pituitary MRI indicating a partially empty sella (red arrow). **(C)** Brain MRI demonstrating diffuse leptomeningeal enhancement (red arrow), consistent with infectious meningitis.

Flow cytometry of CSF identified a total of 500 nucleated cells, predominantly T lymphocytes with no detectable CD19^+^ B lymphocytes. The T-helper to T-cytotoxic cell ratio (CD4^+^/CD8^+^ or T4/T8) was 1.56, within normal range. Electrocardiogram (ECG) demonstrated sinus tachycardia. Echocardiography revealed left atrial enlargement, with mild mitral and tricuspid regurgitation. Abdominal and urinary tract ultrasounds showed no abnormalities. Chest CT showed bilateral pulmonary infiltrates (red arrow), characterized multifocal ground glass opacities and intertistial thickening and multiple enlarged mediastinal, supraclavicular, and axillary lymph nodes. Sagittal contrast MRI of the pituitary (suggested a partially empty sella (red arrow), suggesting pituitary flattening along the sellar floor. Brain MRI (plain, diffusion-weighted imaging [DWI], contrast-enhanced, and MRA) showed diffuse leptomeningeal enhancement, indicating a likely infectious etiology consistent with meningitis (**Figure 1**). No significant abnormalities were observed on cranial MRA or susceptibility-weighted imaging (SWI).

The patient had a prolonged course of recurrent fever and joint swelling, later developing seizures and signs of meningeal irritation. Laboratory investigations revealed leukocytosis and markedly elevated serum ferritin. Bone marrow aspiration, lymph node biopsy, PET-CT (performed externally), and CSF flow cytometry showed no evidence of malignancy. Investigations for tuberculosis and viral infections were unremarkable. Autoantibody panels and autoimmune encephalitis antibodies were also negative, with no evidence supporting other autoimmune diseases. According to the Yamaguchi criteria, the patient fulfilled three major criteria (fever ≥39°C, arthralgia, leukocytosis ≥15×10^9^/L) and two minor criteria (lymphadenopathy, negative RF and ANA), which supported the diagnosis of AOSD after exclusion of other possible cause.

Brain MRI revealed leptomeningeal enhancement. Further tests, including mNGS, autoimmune encephalitis antibodies, and flow cytometry, ruled out autoimmune encephalopathy, intracranial infection, and neoplastic processes supporting CNS involvement secondary to AOSD. The patient also presented with refractory electrolyte disturbances (hyponatremia and hypochloremia) and endocrine abnormalities. After consultation with endocrinology, empty sella syndrome with secondary adrenal insufficiency and hypogonadism was diagnosed.

She was treated with intravenous methylprednisolone (80 mg/day) for approximately one week in March 2024, followed by 60mg/day or nearly two weeks and then 40 mg/day for another 10 days. The dose was gradually tapered from June 2024 afterward, the patient has been maintained on methylprednisolone 8mg once daily. During hospitalization, she also received appropriate antimicrobials for pulmonary infection and antiepileptics per neurology. Antiviral therapy was initiated for active hepatitis B infection.

After pulmonary infection was controlled, the patient underwent three sessions of lumbar puncture with intrathecal injections dexamethasone 10 mg + methotrexate 10 mg weekly). Subsequently, she received immunosuppressive therapy with cyclosporine and tocilizumab. Clinical symptoms gradually improved—consciousness returned to normal, fever subsided, joint pain and fatigue were relieved. Laboratory and imaging results showed improvement. The patient’s condition stabilized, and she was discharged in good condition.

**Figure 2:**
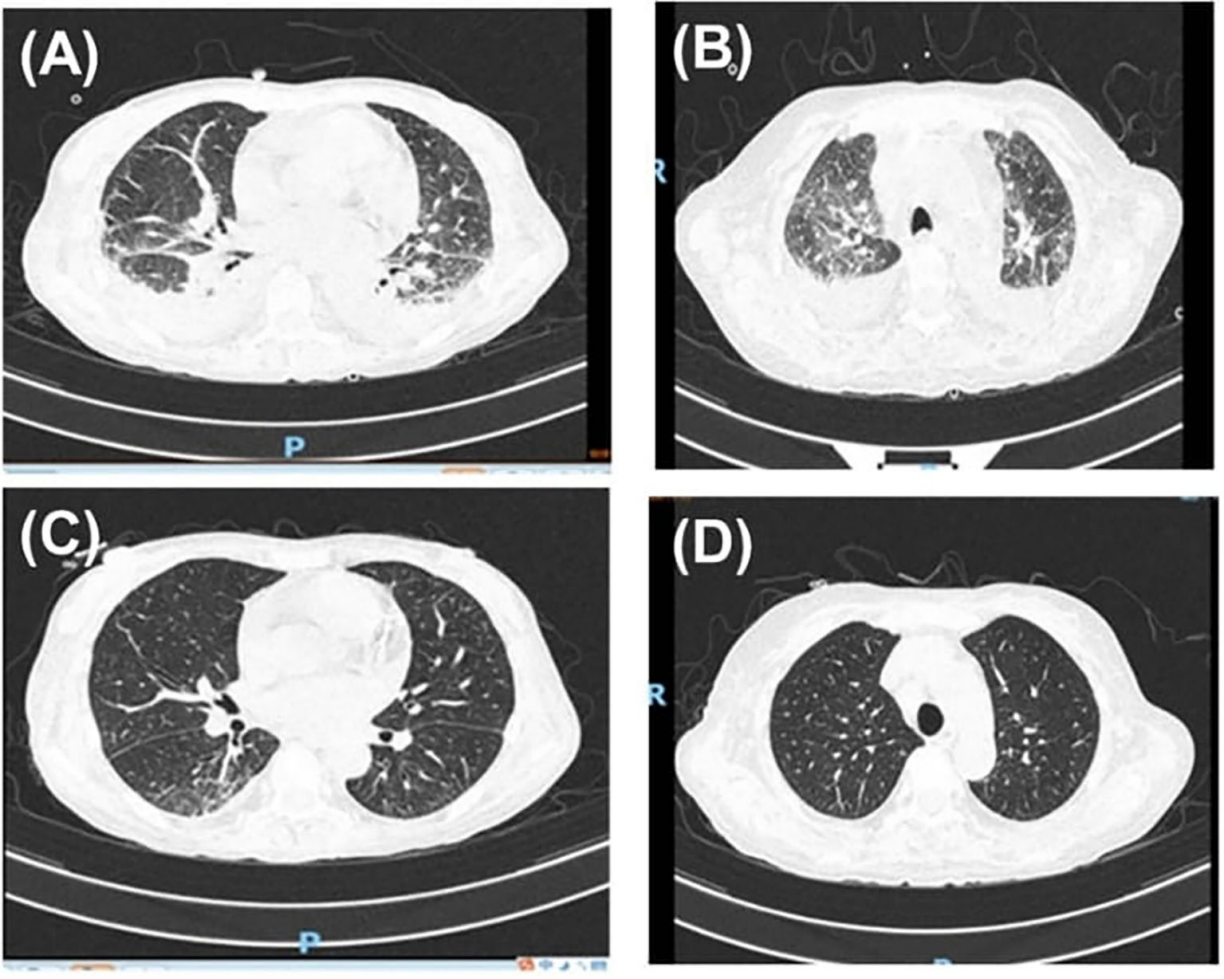
Radiological comparison of chest and brain before and after treatment. **(A, B)** Chest CT obtained in March 2024, prior to treatment showing bilateral pulmonary infiltrates with multifocal ground-glass opacities. **(C, D)** Chest CT obtained in April 2024, after treatment demonstrating interval improvement of the pulmonary infiltrates.

**Figure 3:**
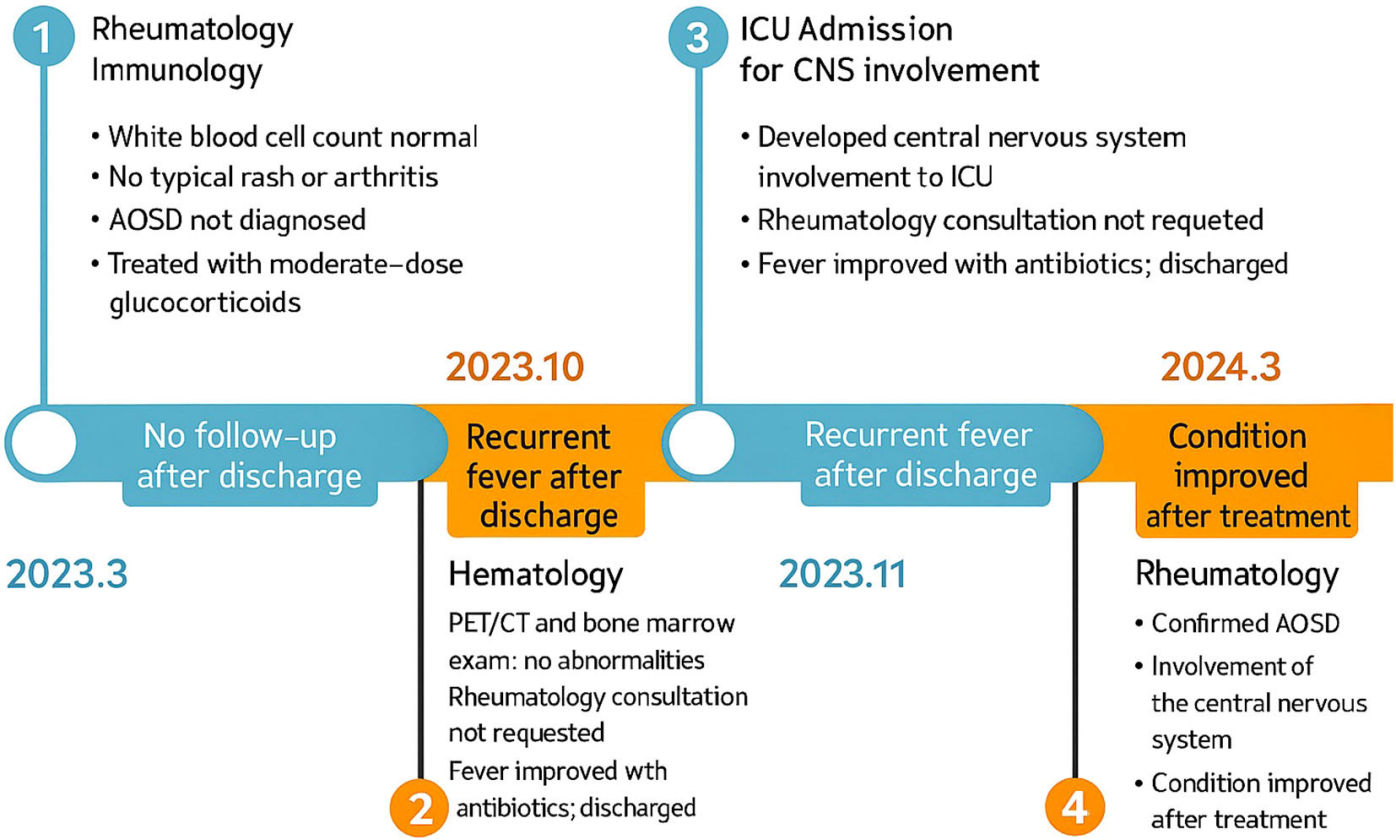
Timeline figure.

After discharge, the patient continued treatment regularly. Prednisone was gradually tapered to 10 mg once daily. Tocilizumab was discontinued after completing six full treatment cycles, while cyclosporine therapy was continued for immunosuppression. Follow-up evaluations demonstrated significant improvement in laboratory parameters, including hemoglobin, serum sodium, and ferritin levels, as well as in chest CT and brain MRI (**Figure 2**). She was first evaluated in March 2023, diagnosed with AOSD in March 2024, and remained clinically stable during follow-up until April 2025 (**Figure 3**).

## Discussion

AOSD presents with a wide spectrum of clinical manifestations, with symptoms varying according to the organs involved. The most common features include quotidian fever, evanescent rash, arthralgia or myalgia, and sore throat. The most frequently affected sites are the skin and joints (2–4). Among the less common but clinically important manifestations is neurological involvement which has been reported in approximately 7% to 12% of AOSD cases (4). Some studies suggest that AOSD patients with neurological involvement may have more severe disease features, including higher ferritin and LDH levels and a higher incidence of MAS (1, 4, 5).CNS involvement may occur either in the early stage of the disease or during its progression.

According to a multicenter study from Japan, among 89 patients with AOSD, 11 exhibited neurological symptoms, with 7 patients diagnosed with CNS involvement. Of these, 4 presented with aseptic meningitis, accounting for approximately 36% of CNS manifestations (6) Several case reports have indicated that CNSinvolvement is predominantly characterized by demyelinating encephalopathy and aseptic meningitis (7–9).

AOSD patients with concomitant aseptic meningitis may present with neurological signs and symptoms such as headache, nausea, vomiting, limb weakness, and positive meningeal irritation signs. They may also exhibit extra-neurological features including rash, sore throat, and lymphadenopathy (1, 4).Laboratory findings often reveal elevated CSF protein levels, leukocytosis, markedly increased serum ferritin, a decreased glycosylated ferritin ratio, and negative RF and ANA (4, 10). The pathogenesis of CNS involvement secondary to AOSD remains incompletely understood. Several possible mechanisms have been proposed: (1) CNS manifestations may represent a component of the systemic inflammatory profile characteristic of AOSD; (2) the marked neutrophilia seen in peripheral blood in AOSD may lead to infiltration of activated neutrophils across the blood-brain barrier, contributing to the development of meningitis; and (3) Domingues et al. hypothesized that coagulopathy and thrombotic microangiopathy may underlie CNS injury in AOSD patients, although this theory still requires further clinical validation (11–13).

In contrast, pulmonary involvement, though recognized as a systemic manifestation of AOSD, is less common than dermatologic or articular symptoms. Chest CT in our patient revealed bilateral pulmonary infiltrates and multiple enlarged mediastinal, supraclavicular, and axillary lymph nodes. These findings likely represent inflammatory changes related to AOSD rather than infection or malignancy, suggesting that pulmonary and lymphatic abnormalities can occur as part of the systemic inflammatory process of this disease (4).

To establish the diagnosis of AOSD, extensive investigations were performed to exclude infectious, autoimmune, and malignant disorders. Multiple blood cultures, respiratory pathogen nucleic acid tests, and viral DNA tests for cytomegalovirus and Epstein–Barr virus were all negative. Tuberculosis screening (PPD and T-SPOT) also yielded negative results, and CSF analyses—including culture, mNGS, and autoimmune encephalitis antibody panels—showed no evidence of infection or autoimmune encephalitis. Autoimmune markers, including ANA, ANCA, ENA panel, RF, and anti-CCP antibody, were negative, effectively ruling out systemic lupus erythematosus, vasculitis, and rheumatoid arthritis. Imaging studies revealed no evidence of malignancy. The combination of quotidian fever, evanescent rash, arthritis, lymphadenopathy, hyperferritinemia (>33,511 ng/mL), and elevated inflammatory markers, in the absence of infection, autoimmune disease, or malignancy, fulfilled Yamaguchi’s criteria and supported the diagnosis of AOSD. According to the Yamaguchi classification criteria, the patient fulfilled three major and four minor criteria. The major criteria included persistent high fever (>39.0°C) lasting for more than one week, arthritis involving both knees and wrists with synovitis confirmed by ultrasonography, and leukocytosis (WBC ≥ 10 × 10^9^/L). The minor criteria included sore throat, abnormal liver function tests, negative rheumatoid factor (RF) and antinuclear antibody (ANA) results, and lymphadenopathy with malignancy excluded. These findings further supported the classification of adult-onset Still’s disease. However, there are currently no specific biomarkers indicative of CNS involvement in AOSD. Therefore, in suspected cases, early lumbar puncture and cranial MRI are essential for differential diagnosis.

In this case, the patient was initially suspected of having an autoimmune inflammatory disorder at her first medical visit. However, due to poor treatment adherence and delayed initiation of appropriate therapy, the disease progressively worsened. During subsequent hospitalizations, she was not referred to a rheumatology specialist, which led to a missed diagnosis of AOSD. As a result, the patient did not receive timely and standardized treatment, leading to disease progression, secondary severe CNS involvement, and concurrent pulmonary infection.

Another notable feature in this case was the presence of persistent hyponatremia, which potentially misled the diagnostic process. Common endocrine-related causes of hyponatremia include hypopituitarism, primary thyroid or adrenal insufficiency, and syndrome of inappropriate antidiuretic hormone secretion (SIADH).In this case, pituitary MRI revealed findings consistent with empty sella syndrome and hormonal testing suggested secondary adrenal insufficieny, pointing towards pituitary dysfunction as the likely mechanism. Given the coexistence of aseptic meningitis, pituitary involvement secondary to AOSD-related inflammation was considered a plausible explanation for the electrolyte disturbance.

Hyponatremia has been frequently reported in CNS diseases and meningitis, most commonly due to SIADH or cerebral salt wasting (14, 15). However, hyponatremia occurring together with empty sella and biochemical evidence of hypopituitarism has rarely been described, and, to our knowledge, no previous reports have documented this constellation in AOSD (15, 16). While AOSD-associated aseptic meningitis (8) and, separately, hyponatremia related to renal tubular dysfunction have been reported (17), the combination of AOSD-related meningitis, empty sella, and pituitary dysfunction has not been previously highlighted. This suggests that our case may represent a unique endocrine–neurological manifestation of AOSD and underscores the importance of evaluating hypothalamic–pituitary function in patients presenting with CNS involvement and unexplained hyponatremia.

According to current case reports, most patients with AOSD have a favorable prognosis with active treatment. However, those with multi-organ involvement, such as secondary MAS or CNS involvement, have significantly higher mortality rates (18). The primary goals of AOSD treatment include controlling clinical symptoms, minimizing target organ damage, and slowing disease progression (19). Management of CNS manifestations should be accompanied by active treatment of the underlying disease (19).

Glucocorticoids remain the most widely used therapeutic agents in clinical practice. They are effective in controlling clinical symptoms in approximately 60% of AOSD patients, with systemic symptoms showing a more significant response compared to articular manifestations. For patients with multisystem involvement, inadequate response to glucocorticoids, or disease relapse upon dose tapering, early initiation of disease-modifying antirheumatic drugs (DMARDs) is recommended (20).Studies have shown that cyclosporine A is particularly beneficial in AOSD patients with hepatic dysfunction and/or macrophage activation syndrome (MAS), facilitating earlier control of disease activity. AOSD is often associated with elevated levels of inflammatory cytokines such as TNF-α, IL-1β, IL-6, and IL-18, which are believed to play a critical role in the pathogenesis and progression of the disease (21). An increasing number of studies suggest that cytokine-targeted inhibitors can effectively suppress the inflammatory response and alleviate clinical symptoms.

In this case, the patient achieved a favorable systemic response to the combination therapy consisting of glucocorticoids, cyclosporine A, and the IL-6 inhibitor tocilizumab, resulting in disease improvement within two weeks. Patients with AOSD often exhibit elevated levels of multiple inflammatory cytokines, including TNF-α, IL-6, IL-1, and IL-18, all of which contribute to disease pathogenesis and progression (22). Numerous studies have demonstrated that inhibitors targeting IL-6, IL-1, or TNF-α can effectively suppress the inflammatory response and improve clinical outcomes in AOSD (23). Given that this case represented severe AOSD with central nervous system involvement, biologic therapy was indicated. However, only IL-6 antagonists tocilizumab and TNF-α inhibitors adalimumab were available at our institution. Considering the patient’s risk of infection, tocilizumab was preferred. From the perspective of AOSD pathogenesis, IL-1 blockade would also be an appropriate therapeutic option in patients with severe disease.

However, because she had confirmed AOSD with central nervous system involvement and presented with a concomitant infection at the time of admission in March 2024, high-dose glucocorticoid pulse therapy was considered inappropriate. Although methotrexate is a potent immunosuppressive agent, its penetration across the blood–brain barrier is poor. Drawing on therapeutic experience from central nervous system leukemia (24), intrathecal combination therapy was therefore selected to achieve high local drug concentrations within the CNS and rapidly alleviate AOSD-related aseptic meningitis. The intrathecal administration of corticosteroids and methotrexate likely contributed to the rapid improvement of the patient’s neurological manifestations. This route of delivery allows anti-inflammatory agents to reach higher concentrations within the cerebrospinal fluid, thereby providing targeted suppression of meningeal inflammation, as reported in previous cases of AOSD with severe neurological involvement (1, 25, 26). The patient subsequently maintained long-term clinical remission.

## Conclusion

Given the complexity of AOSD with neurological involvement, clinicians should maintain a high index of suspicion for AOSD in patients presenting with fever, rash, and joint pain accompanied by neurological symptoms. Early and comprehensive diagnostic evaluation, including appropriate laboratory and imaging studies, is essential for confirming the diagnosis and excluding other potential causes. Prompt initiation of targeted treatment is critical for improving prognosis and reducing the risk of misdiagnosis or treatment delay.

## Data Availability

All relevant data supporting the findings of this study are contained within the article. No additional datasets were generated or analyzed for this study.
