# Supplementary figures and images for: Adult-onset Still’s disease presenting with aseptic meningitis: a case report
Source: Front Immunol. 2025 Dec 16;16:1660434. doi: 10.3389/fimmu.2025.1660434 (PMC12747900; doi:10.3389/fimmu.2025.1660434)

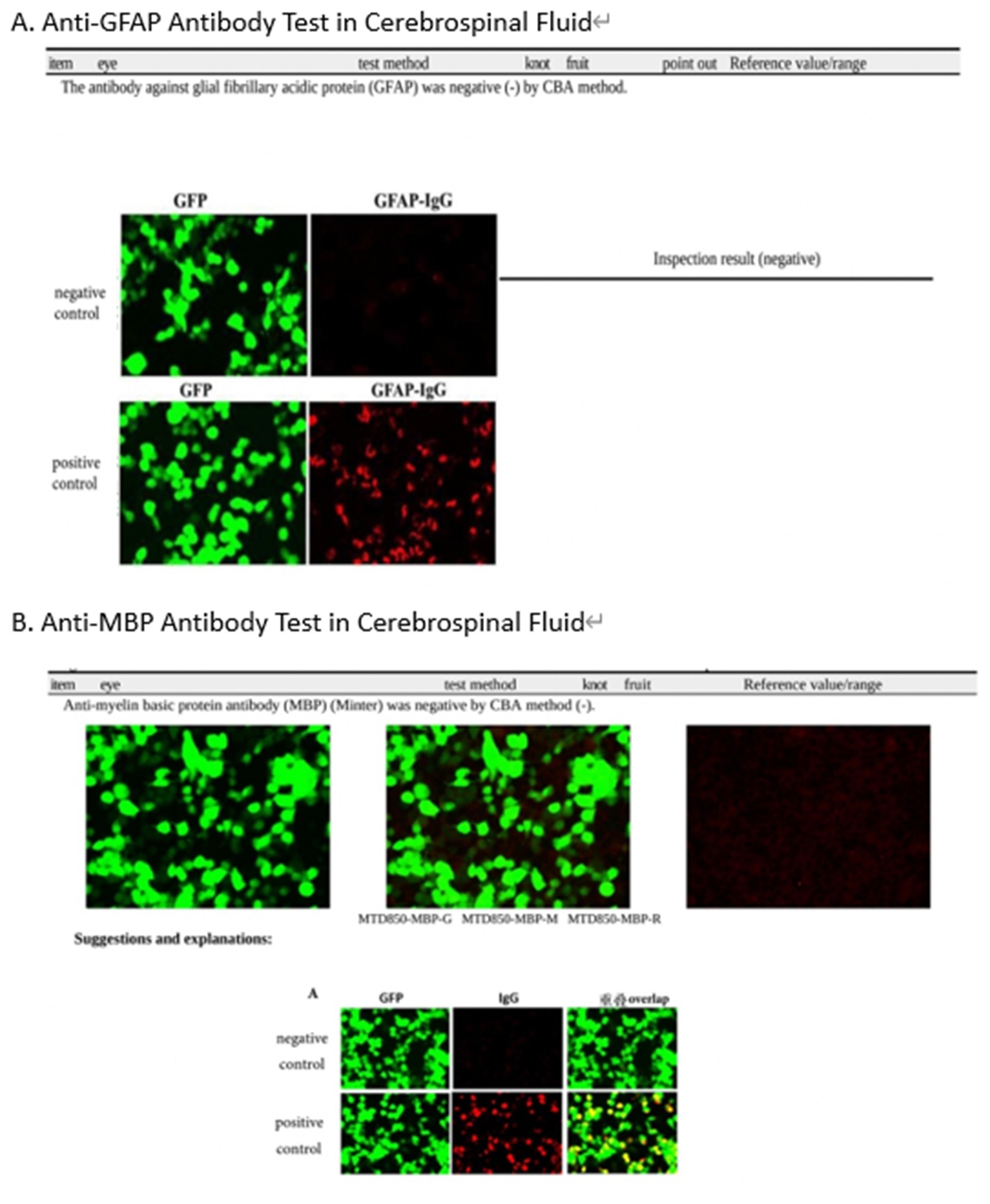

Supplement: Supplementary file 2 [file Image1.jpeg]
